# Autoamputation and Polyneuropathy in Mixed Connective Tissue Disorder: A Case Report

**DOI:** 10.7759/cureus.1313

**Published:** 2017-06-05

**Authors:** Syeda Naqvi, Vikash Talib, Razia Aijaz, Zeeshan Ali, Shehroz Bashir, Syed Masroor Ahmad, Shabnam Naveed

**Affiliations:** 1 Jinnah Postgraduate Medical Centre, Jinnah Sindh Medical University (SMC); 2 Accident & Emergency, Jinnah Postgraduate Medical Center Karachi Pakistan; 3 Internal Medicine, Jinnah Postgraduate Medical Center Karachi Pakistan; 4 Jinnah Postgrduate Medical Centre, Jinnah Sindh Medical University (SMC); 5 Medicine Unit 7, Jinnah Postgraduate Medical Center Karachi Pakistan; 6 Medicine, Jinnah Postgraduate Medical Centre Karachi Pakistan; 7 Department of Medicine, Jinnah Postgraduate Medical Center Karachi Pakistan

**Keywords:** mixed connective tissue disorder, autoamputation, polyneuropathy, sensorimotor neuropathy, raynauds, sclerodactyly, calcinosis

## Abstract

Mixed connective tissue disorder (MCTD) is a multisystem disease with overlapping features of other autoimmune diseases, such as systemic lupus erythematosus (SLE), myositis, rheumatoid arthritis, and scleroderma. MCTD presents with a distinctive antibody in serum known as U1-ribonucleoprotein (RNP). MCTD is quite rare as compared to other connective tissue disorders like SLE, systemic sclerosis, dermatomyositis, and polymyositis. We describe a case of MCTD in a young Asian female of 30 year old. This case highlights rare co-existence of polyneuropathy and autoamputation in MCTD disorder. Trigeminal neuralgia and cranial nerve involvements have been previously reported in MCTD but the findings of polyneuropathy and autoamputation are extremely rare.

## Introduction

Mixed connective tissue disease (MCTD), first described in 1972 by Sharp, represents an overlapping disease of different autoimmune conditions such as systemic lupus erythematosus (SLE), scleroderma, rheumatoid arthritis, polymyositis, and dermatomyositis that often exist with antibodies targeted towards the U1 small nuclear ribonucleoprotein (RNP) autoantigen [1]. MCTD or Sharp syndrome is usually considered incurable and presents with a broad spectrum of symptoms such as Raynaud's phenomenon, swollen fingers, erosive arthritis, sclerodactyly along with pulmonary and central nervous system involvement [2].

MCTD is diagnosed on clinical and serologic findings and usually responds to low-dose glucocorticoids. Here we present a unique case of MCTD with polyneuropathy along with auto-amputation of a tip of her finger. Informed consent statement was obtained for this study.

## Case presentation

**Figure 1:**
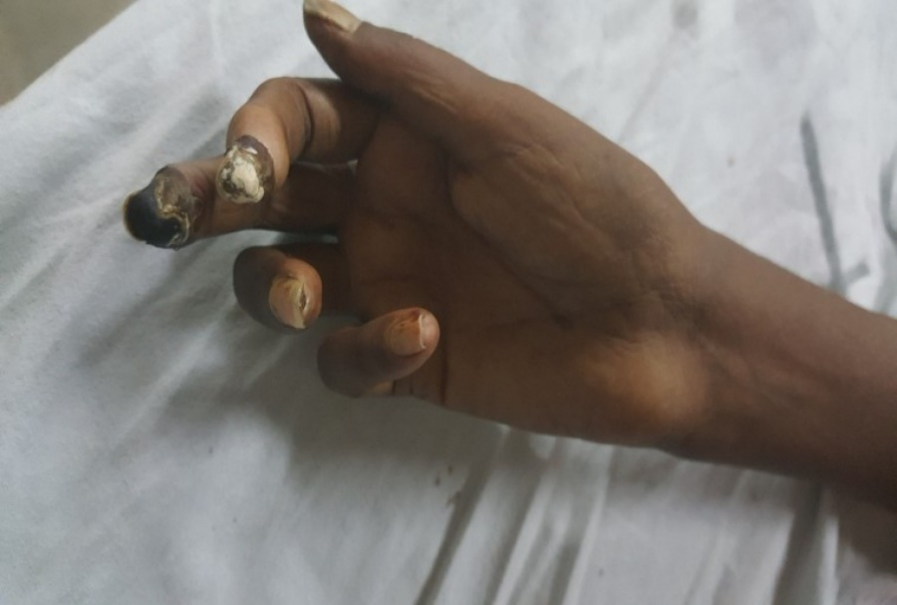
Acral ulcers

A 30-year-old female, married with no known prior co-morbidities, was admitted with complaints of black discoloration of fingers for two years, joint pain for six months, and lower limb weakness for three months. She was in her usual state of her health two years back when she suddenly developed a small, painful area of black discoloration over the tip of her left index finger that gradually increased and later her left ring finger was also involved. Her left index finger underwent autoamputation after two months of the development of the discoloration as showed in Figure 1. Four months back she developed similar complaints in her right index and middle fingers. She has a history of blue discoloration of her fingers on exposure to water for last two years. It was associated with cold sensation and numbness. Her fingers became red and tender after warming.

For the past six months, she had multiple joint pains, starting from small joints of her hands and elbows bilaterally. Pain is moderate to severe in intensity, aggravated by movement with no special time of occurrence or morning stiffness and not associated with redness or swelling; relieved by medications. She developed bilateral lower limb weakness three months back which was of gradual onset and progressive in nature; associated with numbness in both feet. She was initially able to walk with support, but for the past two weeks now she has become bed bound.

She also had easy fatigability and shortness of breath on exertion which was progressive in nature. There was no history of chest pain, palpitations, paroxysmal nocturnal dyspnea, or orthopnea. She had lost weight significantly over the period of six months that is undocumented and unintentional while her appetite had been normal. There is no history of fever, dry eyes, rashes, oral ulcers, photosensitivity, alopecia, epilepsy, difficulty in swallowing, hearing problem, double vision, frothing of urine and abortions or miscarriages; no history of any other systemic chronic illness; and no history of surgeries or blood transfusions. She had been taking multiple over-the-counter painkillers and multivitamin supplements. Her gynecological and obstetrical history was not significant. She had an eight-year-old son, alive and healthy. Her family history was also non-contributory. She was addicted to betel nuts, taking it almost 10-12 times a day.

**Figure 2:**
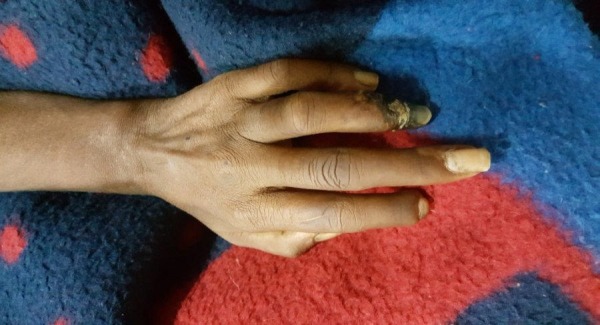
Sclerodactyly and ulceration of fourth finger

On physical examination, a young female of average height and built, lying comfortably on the bed, was well oriented to time, place and person. She had pale, thick, and shiny skin over the face. She had restricted mouth opening and taut skin over oral fissure bilaterally. She had sclerodactyly over both hands and calcinosis over the tips of the right index, middle, ring fingers and left ring finger as shown in Figure 2. Gangrenous distal phalanges of the index and middle fingers of the right hand and ring finger of the left hand were found. There was auto-amputation of distal phalanx of left index finger. Her small joints of the hand (metacarpophalangeal, proximal interphalangeal and distal interphalangeal joint) examination showed flexion contractures at the proximal interphalangeal joints of both hands. No erythema, swelling, or muscle wasting was noted. There was mild tenderness over proximal interphalangeal joints and restricted extension at proximal interphalangeal and distal interphalangeal joint due to pain. Her left elbow was swollen and was held in a flexed position and restricted movements in extension. There was a restriction of extension at 115 degrees due to pain. Her central nervous system examination showed a decrease in power in both lower limbs (2/5). She had loss of sensations below the knee bilaterally. The rest of the examination was unremarkable.

**Figure 3:**
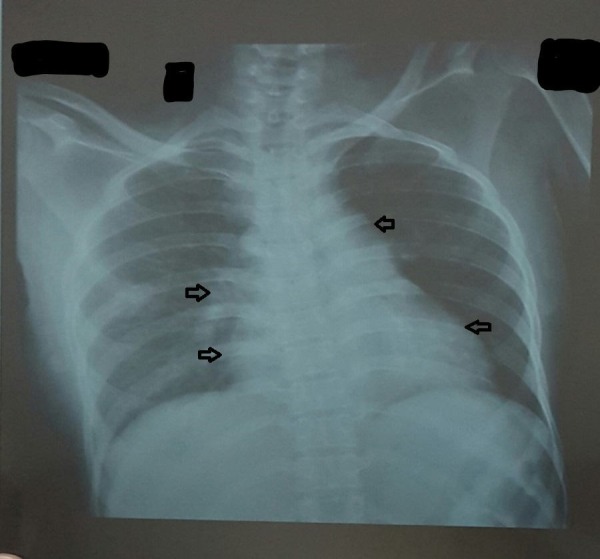
Chest x-ray Arrows pointing towards cardiomegaly.

Her baselines including complete blood count, basic metabolic panel, liver function test, urine detailed report, renal function test, chest x-ray, echocardiography, computed tomography (CT) scan of the brain, ultrasound abdomen, and creatinine phosphokinase (CPK) levels were ordered. Her test as shown in Table 1 showed that she has microcytic anemia with the raised erythrocyte sedimentation rate (ESR). Echo showed grade 1 diastolic dysfunction. Chest x-ray showed cardiomegaly as seen in Figure 3. Her CPK levels were 1027IU/L (normal range = 22 to 198 units per liter). Her electromyography (EMG) showed symmetric distal axonal sensorimotor neuropathy. Partial conduction block due to axonal degeneration was seen and no demyelination was observed. The patient denied a muscle biopsy.

**Table 1:** Baselines

Labs	Values	Labs	Values
Hemoglobin	8.5 g/dl	Urea	21 mg/dl
Hematocrit	26.6%	Creatinine	0.7 md/dl
Mean cell volume	62.7%	Sodium	133 mEq/L
Mean cell hemoglobin	19 pg	Potassium	3.4 mEq/L
Platelet count	317 x 10^9^ /L	Chloride	100 mEq/L
White blood cell count	15 x 10^9^/L	Activated prothrombin time	23.9 sec
Neutrophils	75%	Prothrombin time	9.3 sec
Lymphocytes	18%	International normalized ratio	0.89
Eosinophils	04%	Total bilirubin	0.38 mg/dl
Monocytes	03%	Direct bilirubin	0.14 mg/dl
Gamma glutamyltransferases	20 U/L	Alkaline phosphatase	168 U/L
Erythrocyte sedimentation rate	120 mm/hr	Alanine aminotransferases	62 U/L
C-reactive protein	52.9	Albumin	2.6 g/dl

 

Serological examination showed anti-nuclear antibody, anti-U1 RNP antibody, anti-topoisomerase antibodies (ScL-70), anti-Ro and anti-La antibodies came positive. However, anti-smooth muscle and antimitochondrial antibody came back negative. The diagnosis of mixed connective tissue disorder was made.

She was started on Prednisolone 25 mg and Cilostazol 100 mg twice a day, tablet omeprazole 40 mg, Spironolactone 300 mg, Injection Amikacin 500 mg, Aspirin 75 mg once a day was given for two weeks. The patient was discharged after two weeks. She was followed up every month since then and after four months, the patient recovered from most of her complaints except lower limb weakness which persists even after treatment.

## Discussion

Mixed connective tissue disease, an autoimmune condition, which may affect almost any organ system of the body, presents with overlapping features from major connective diseases such as SLE, scleroderma, rheumatoid arthritis, dermatomyositis, and polymyositis along with an antibody against the RNP antigen [1]. Recently, a study from Norway showed an annual incidence to be 2.1 per million per year and points prevalence to be 3.8 per 100,000 indicating that MCTD is the least common connective tissue disorder [2].

Three different classifications to assess MCTD exist: Kasukawa, Alarcon-Segovia, and Sharp. In a retrospective study of 161 patients, Cappelli, et al., showed that Kasukawa criteria were most sensitive in assessing MCTD with a sensitivity of 75%. Alarcon-Segovia and Sharp had a diagnostic sensitivity of 73 and 42%, respectively [3]. We diagnosed our patient based on Kasukawa criteria.

Most patients present with typical findings of Raynaud's phenomenon, swollen fingers, arthralgia, myalgia along with pulmonary, cardiovascular, and central nervous system involvement [2]. Our patient also presented with symptoms of Raynaud's phenomenon and arthritis, but secondary Raynaud’s phenomenon had progressed to acral ulcers and the patient underwent auto-amputation of left index finger. The proposed treatments of acral ulcer are iloprost, hyperbaric oxygen, sympathetic block, and analgesics [4].

Other than dermatological findings, another purpose of this case report was to enlighten the neurological findings associated with mixed connective tissue disorder. Previously, conditions like trigeminal neuralgia, bilateral optic neuropathy, and transverse myelopathy have been seen in concordance with mixed connective tissue disease [5-6]. Another case of subacute progressive encephalopathy was described in mixed connective tissue disease secondary to vasculitis of central nervous system [7]. Bilateral facial nerve palsy with facial swelling can also present with mixed connective tissue disorder known as Melkersson-Rosenthal syndrome [8]. Neuropathy is one of the common neurological manifestations of MCTD. Neuropathies related to MCTD can be of three types, i.e., vasculitic, non-vasculitic, or treatment-related neuropathy [9].

Vasculitic neuropathy can be confluent and present as distal symmetric neuropathy. Distal symmetric neuropathy can also be of no-vasculitic origin. Compressional neuropathies like carpal tunnel syndrome can also be seen [10]. Neuropathies often herald the diagnosis of mixed connective tissue disorder. In our case, the patient presented with severe Raynaud’s, amputation and polyneuropathies.

MCTD is associated with many complications affecting different organ systems. There is no specific principal regarding the treatment of MCTD but the consensus is that corticosteroids are the most beneficial [2,4]. Other drugs that help patients with MCTD, targeting various symptoms associated with the disease, include azathioprine, nifedipine, nitroglycerin, and tumor necrosis factor blockers, etc.

## Conclusions

Neurological involvement is common among patients with MCTD. Prompt non-invasive tests like nerve conduction studies and clinical diagnosis might have a role in screening for early diagnosis and timely treatment. Mixed connective tissue disease should be considered as an important syndrome in any patient who presents with complex clinical findings, including neuropathies and who do not fit into any defined criteria of systemic connective tissue disorders.
